# Asian G6PD-Mahidol Reticulocytes Sustain Normal *Plasmodium Vivax* Development

**DOI:** 10.1093/infdis/jix278

**Published:** 2017-06-07

**Authors:** Germana Bancone, Benoit Malleret, Rossarin Suwanarusk, Nongnud Chowwiwat, Cindy S Chu, Rose McGready, Laurent Rénia, François Nosten, Bruce Russell

**Affiliations:** 1 Shoklo Malaria Research Unit, Mahidol-Oxford Tropical Medicine Research Unit, Faculty of Tropical Medicine, Mahidol University, Mae Sot, Thailand;; 2 Singapore Immunology network (SIgN), A*STAR, 8A Biomedical Grove, Singapore 138648, Singapore;; 3 Department of Microbiology and Immunology, Yong Loo Lin School of Medicine, National University of Singapore, National University Health System, 5 Science Drive 2, Blk MD4, Level 3, Singapore 117597, Singapore;; 4 Department of Microbiology and Immunology, University of Otago, Dunedin, New Zealand;; 5 Centre for Tropical Medicine and Global Health, Nuffield Department of Medicine Research building, University of Oxford Old Road campus, Oxford, UK.

**Keywords:** glucose-6-phosphate dehydrogenase deficiency, *Plasmodium vivax*, reticulocytes

## Abstract

Glucose-6-phosphate dehydrogenase (G6PD) deficiency is the most common enzymatic disorder in humans and appears to be protective against falciparum severe malaria. Controversially, it is also thought that *Plasmodium vivax* has driven the recent selection of G6PD alleles. We use an experimental approach to determine whether G6PD-Mahidol^G487A^ variant, a widespread cause of severe G6PD deficiency in Southeast Asia, provides a barrier against vivax malaria. Our results show that the immature reticulocytes (CD71^+^) targeted by *P. vivax* invasion are enzymatically normal, even in hemizygous G6PD-Mahidol ^G487A^ mutants; thus, allowing the normal growth, development, and high parasite density in severely deficient samples.

Mutations in the X-linked glucose-6-phosphate dehydrogenase (*G6PD*) gene are the most common and widespread cause of human enzymopathy; they are associated with stress-induced hemolysis, resulting in a range of mild to life-threatening clinical conditions. The degree of phenotypic G6PD deficiency (G6PDd) depends on the genetic variant and the zygosity of the carrier [1], and it is most commonly due to an instability of the G6PD enzyme [2] or an altered functionality of the protein active site expressed in mature erythrocytes [3].

G6PDd is protective against severe malaria caused by *Plasmodium falciparum* [4] as documented by epidemiological evidence [5] and in vitro studies on parasite development [6]. Recent studies suggest that by reducing levels of parasitemia, the selection of *G6PD* mutations has also been driven by *P. vivax*, the other major cause of human malaria [7]. However, this is in contradiction to an earlier study showing higher *P. vivax* parasitemias in G6PDd young children as compared to G6PD normal [8] in the same population. Here, we conduct the first ever ex vivo experiments to determine whether the G6PD-Mahidol mutation (487G>A) in hemizygous and homozygous status poses a barrier to the invasion and the normal development of vivax malaria parasites.

## EXPERIMENTAL PROCEDURES

### Ethics Statement

The clinical infected red blood cell samples examined in this study were collected under the following ethical guidelines in the approved protocols: OXTREC 45–09 (University of Oxford, Centre for Clinical Vaccinology and Tropical Medicine, UK) and MUTM 2008–215 (Ethics Committee, Faculty of Tropical Medicine, Mahidol University, Thailand).

### G6PD Characterization

Blood samples were collected at the Thailand–Myanmar border among subjects of Karen ethnicity where the prevalence of G6PD can reach 15%; the major variant found in the population is Mahidol (487G>A) (Supplementary Figure 1).

Blood samples were screened by fluorescent spot test (R&D Diagnostics, Greece) and genotyped for the Mahidol variant using a polymerase chain reaction–restriction fragment length polymorphism protocol [9]. Quantitative phenotypic characterization of samples was performed by gold standard spectrophotometric assay using Trinity Kits (Trinity, Ireland) and flow-cytometric assay according to Shah et al. [10].

### Human Parasites

Thirty-four clinical isolates of *P. vivax* were collected from malaria patients receiving treatment at clinics run by the Shoklo Malaria Research Unit on the northwestern border of Thailand. The project was explained to all the patients before they provided informed consent prior to collection of blood by venipuncture. Whole blood (5 mL) was collected in lithium heparin collection tubes. These samples were cryopreserved in Glycerolyte 57 Solution (Baxter) after leukocyte depletion using a nonwoven fabric filter (Antoshin Pte Ltd). After thawing, the parasites present in the packed cells (1.5 mL per isolate) were cultured to the schizont stage in 12 mL McCoy 5A medium supplemented with 2.4 g/L D-glucose, 40 mg/mL gentamycin sulfate, and 20% heat-inactivated human AB serum, in an atmosphere of 5% O_2_ at 37.5°C.

### Immunomagnetic Erythrocyte Sorting

The selection of the CD71^+^ reticulocytes was performed using the magnetic-activated cell sorting system (Miltenyi Biotec). A total of 2 mL blood at 50% hematocrit in phosphate-buffered saline was passed through an LS column (Miltenyi), the purity of the positive and negative fractions was monitored by flow cytometry using Thiazole orange (Sigma-Aldrich) staining. The yield of CD71^+^ cells is upper 90% of purity.

### Scanning Electron Microscopy

Electron microscopy was conducted on reticulocytes and normocytes after immunomagnetic sorting using the methods outlined in Malleret et al. [11].

### Optical Microscopy

Microscopic enumeration of infected red blood cells was performed using thin blood smears stained with Giemsa. A minimum of 4000 red blood cells were counted (20 fields at 100× magnification). Live cell subvital staining of reticulocytes and parasites was done using Giemsa.

### Statistical Analyses

Statistical analyses were performed using Graph Pad Prism (5.1). Mean values were compared, and *P* values were calculated using paired *t* tests.

## RESULTS AND DISCUSSION

Unlike *P. falciparum*, *P. vivax* only invades reticulocytes. Importantly, *P. vivax* targets a specific population of immature reticulocytes (CD71^+^), which are primarily found in the bone marrow [12]. Our hypothesis was that selection of G6PD mutations by *P. vivax* might be mediated by phenotypic deficiency in those young reticulocytes, whereby the parasite would be unable to invade or normally replicate in red blood cells (RBCs) whose intracellular capacity of respond to stress was decreased by lack of G6PD activity.

**Figure 1.**
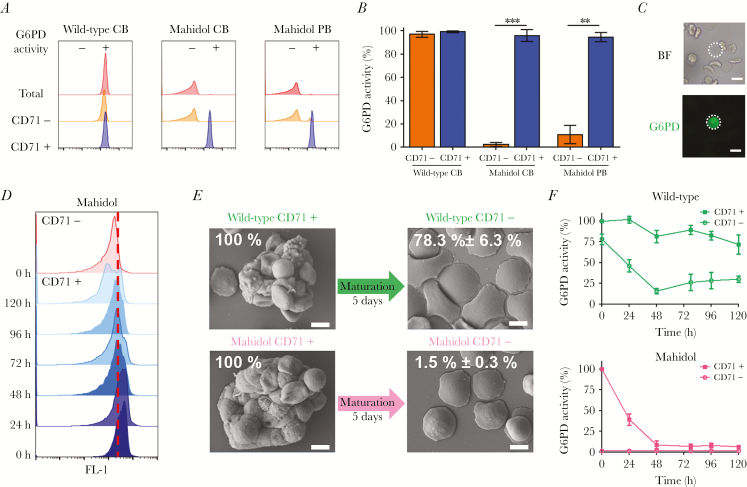
CD71^+^ reticulocytes are phenotypically G6PD normal in subjects with Mahidol Mutation. *A*, G6PD activity detected by flow cytometry in erythrocytes, CD71^–^ and CD71^+^ fraction of wild-type and G6PD-Mahidol mutant cord blood and in G6PD-Mahidol mutant peripheral blood. *B*, Quantification of G6PD activity (mean values ± SDs) for 4 wild-type cord blood, 3 G6PD-Mahidol–deficient cord blood, and 3 G6PD-Mahidol peripheral blood samples. *C*, G6PD activity detected by fluorescence microscopy in CD71^–^ fraction of Mahidol mutant cord blood (scale bar represents 5 μm). *D*, G6PD activity time course detected by flow cytometry in CD71^+^ and CD71^–^ erythrocytes. The red dotted line represents the limit of positivity for G6PD (separation between methemoglobin and oxyhemoglobin peaks of green fluorescence). *E*, Morphology of immunomagnetically sorted CD71^+^ and CD71^–^ erythrocytes from wild-type and G6PD-Mahidol–mutant patients visualized by scanning electron microscopy. Scale bar represents 5 μm. *F*, G6PD kinetic activity in CD71^+^ and CD71^–^ for wild-type and G6PD-Mahidol patients (n = 4) measured by flow cytometry during 5 days of culture at 37.5°C and 5% CO_2_ in McCoy medium 20% AB serum. The MFI of oxyhemoglobin for CD71^+^ reticulocytes at time 0 was assigned to be 100% of G6PD activity for each sample. Abbreviations: G6PD, glucose-6-phosphate dehydrogenase; MFI, mean fluorescence intensity.

We first characterized the G6PD phenotypes of CD71^+^ reticulocytes in blood samples of Karen donors who were either wild-type genotype or hemi/homozygous for the Mahidol mutation. Analyses by flow cytometry and fluorescent microscopy showed that immature reticulocytes have a normal G6PD phenotype, irrespective of the genetic background of the individual (Figure 1A–1C). We studied the biochemical kinetics of immunomagnetically sorted and ex vivo matured CD71^+^ and CD71^–^ erythrocytes from wild-type and G6PD-Mahidol mutant patients and showed that CD71^+^ reticulocytes from hemizygous mutants maintain normal G6PD activity throughout the period that the reticulocyte is receptive to *P. vivax* merozoite invasion [12] (Figure 1D–1F). Upon maturation to normocytes, only genetically wild-type G6PD RBCs retained normal activity. Earlier studies showed that in G6PDd individuals with African mutations, the young erythrocytes produced in response to drug-induced hemolysis had normal enzymatic activity and therefore were not susceptible to hemolysis during the continued administration of oxidative drug [13]; further evidence indicated that G6PD activity was normal in young RBCs of the A variant but very low in young RBCs of the Mediterranean variant [14]. This is the first study to show that CD71^+^ reticulocytes in G6PD-Mahidol mutants have normal G6PD enzymatic activity.

**Figure 2.**
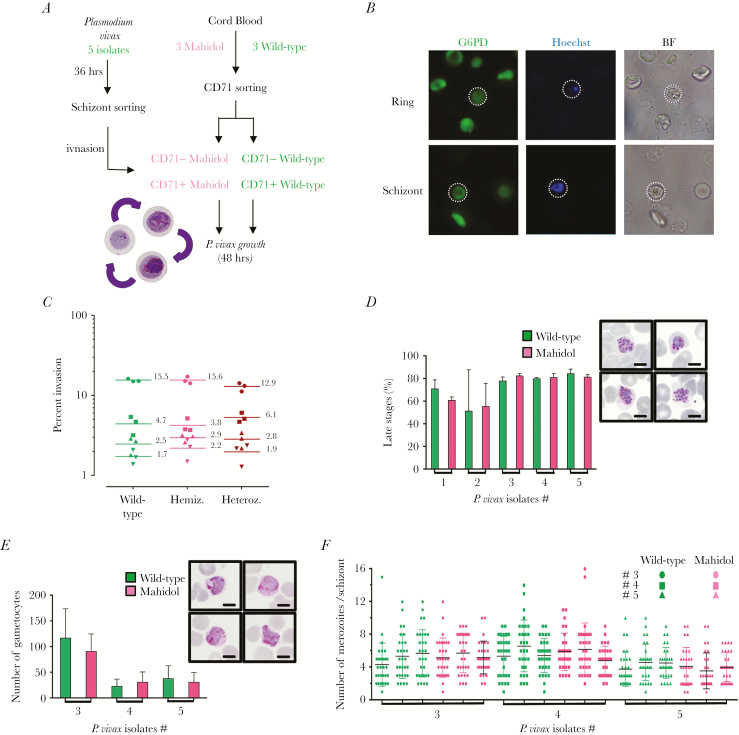
Normal development of *Plasmodium vivax* in G6PD-Mahidol–mutant reticulocytes. *A*, Schematic of *P. vivax* growth assay in G6PD wild-type and G6PD-Mahidol–mutant reticulocytes. *B*, G6PD activity detected by fluorescence microscopy in ring and schizont stages 3 hours and 40 hours, respectively, after inoculation into nascent reticulocytes. *C*, *P. vivax* invasion in adult blood from 3 different genotypes (wild-type, and Mahidol homozygote and heterozygote) with 4 different field isolates. *D*, Proportion of late parasite stages after 48 hours of culture in G6PD wild-type and G6PD-Mahidol mutant (n = 3 for each genotype with 5 different *P. vivax* isolates; 48 hr postinvasion). In insert, Giemsa-stained thin film showing segmented *vivax* schizonts in G6PD-Mahidol–mutant reticulocytes (54 hr postinvasion). The scale represents 5 μm. *E*, *Plasmodium vivax* gametocyte frequency in wild-type and G6PD-Mahidol reticulocytes. In insert, Giemsa-stained thin film showing *vivax* gametocytes in G6PD-Mahidol–mutant reticulocytes (54 hr postinvasion). The scale represents 5 μm. *F*, Number of *vivax* merozoites per schizonts in wild type and G6PD-Mahidol–mutant CD71^+^ reticulocytes (n = 3 for each genotype with 3 different *P. vivax* isolates).

To prove that these Mahidol CD71^+^ reticulocytes are indeed receptive to invasion and capable of supporting the full asexual and sexual erythrocytic life cycle, we conducted a number of ex vivo invasion and maturation assays using freshly isolated *P. vivax* (Figure 2A). Our results show that *P. vivax* merozoites invade Mahidol CD71^+^ reticulocytes equally as well as wild-type CD71^+^ reticulocytes (Figure 2B and 2C), and that full erythrocytic life cycle of *P. vivax* takes place at the same rate (Figure 2D and 2E), resulting in a similar number of merozoites per schizont (Figure 2F). The development of healthy male and female gametocytes occurs in Mahidol CD71^+^ reticulocytes at similar proportions as those seen in G6PD wild-type cells (Figure 2E). Thus, there seems to be no major biological impediment to the development of *P. vivax* in Mahidol CD71^+^ reticulocytes [15]. If there is indeed a protective effect of G6PDd-Mahidol against vivax malaria parasitemia, it may be due to immune host response rather than any intrinsic erythrocytic barriers.

Quite often, it is assumed that malaria has been a major force in driving the evolution of human RBC mutations; while this seems to be the case for *P. falciparum*, the picture is less clear for *P. vivax.* The progressive loss of enzymatic activity upon maturation, especially in G6PD-deficient RBCs but also in the normal ones, might have contributed to shift the preference of plasmodia toward the invasion of younger RBCs. G6PD deficiency might then be among a variety of host mutations that may have driven the selection of parasites that prefer to invade younger RBCs; in particular, reticulocytes.

## Supplementary Data

Supplementary materials are available at *The Journal of Infectious Diseases* online. Consisting of data provided by the authors to benefit the reader, the posted materials are not copyedited and are the sole responsibility of the authors, so questions or comments should be addressed to the corresponding author.

## Supplementary Material

Supplementary-Figure-1

Supplementary_Figure_Legends

## Notes

***Acknowledgements.*** We thank the staff and patients attending the Mae Sot Malaria Clinic, and also clinics associates with the Shoklo Malaria Research Unit (SMRU), Tak Province, Thailand. We thank Daniel Parker for creating the map of Supplementary Figure 1.

***Financial support.*** This work was supported with a Young Investigator grant (BMRC YIG Grant No: 13/1/16/YA/009, to B. M.) under the Agency for Science, Technology and Research (A*STAR, Singapore) and had a joint appointment with the National University of Singapore, funded from the Singapore National Medical Research Council (NMRC/CBRG/0047/2013). L. R. was supported by funding from the Singapore Immunology Network (SIgN) and the Horizontal Programme on Infectious Diseases under the Agency for Science, Technology and Research (A*STAR, Singapore). B. R. was funded by the University of Otago, New Zealand. Shoklo Malaria Research Unit is part of the Mahidol Oxford University Research Unit, supported by the Wellcome Trust of Great Britain. G. B. and C. S. C. were supported by the Wellcome Trust of Great Britain (Programme Grant 089179).

***Potential conflicts of interest.*** All authors: No reported conflicts of interest. All authors have submitted the ICMJE Form for Disclosure of Potential Conflicts of Interest. Conflicts that the editors consider relevant to the content of the manuscript have been disclosed.
