# Supplementary figures and images for: Asian G6PD-Mahidol Reticulocytes Sustain Normal Plasmodium Vivax Development
Source: J Infect Dis. 2017 Jun 7;216(2):263–6. doi: 10.1093/infdis/jix278 (PMC5853331; doi:10.1093/infdis/jix278)

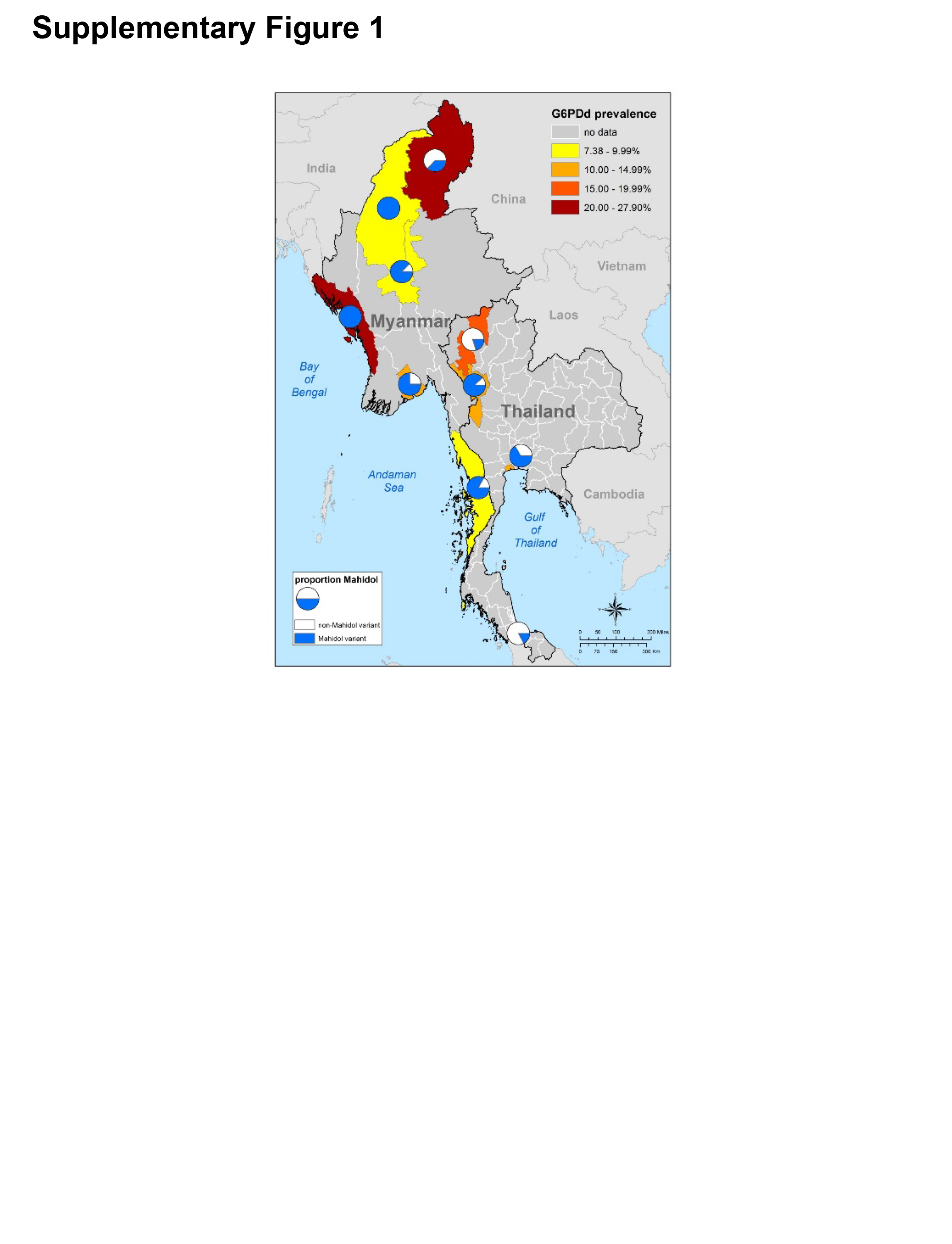

Supplement: Supplementary-Figure-1 [file jix278_suppl_supplementary_figure_1.png]
