# Supplementary material for: Asian G6PD-Mahidol Reticulocytes Sustain Normal Plasmodium Vivax Development
Source: J Infect Dis. 2017 Jun 7;216(2):263–6. doi: 10.1093/infdis/jix278 (PMC5853331; doi:10.1093/infdis/jix278)
Supplement: Supplementary_Figure_Legends [file jix278_suppl_supplementary_data.docx]

**Supplementary Figure Legends**

**Supplementary Figure 1. Map of G6PD Mahidol mutation distribution in different regions of Thailand and Myanmar.** The prevalence of G6PD deficiency is represented by the colour gradient (brown for the highest to yellow for the lowest one). The blue portion of the pie chart is associated with the percentage of Mahidol mutants among all the deficient population. Data from [16-22].

**Supplementary data references**

16. Jalloh A, Tantular IS, Pusarawati S, et al. Rapid epidemiologic assessment of glucose-6-phosphate dehydrogenase deficiency in malaria-endemic areas in Southeast Asia using a novel diagnostic kit. Trop Med Int Health **2004**; 9:615-23.

17. Li Q, Yang F, Liu R, et al. Prevalence and Molecular Characterization of Glucose-6-Phosphate Dehydrogenase Deficiency at the China-Myanmar Border. PLoS One **2015**; 10:e0134593.

18. Nuchprayoon I, Louicharoen C, Charoenvej W. Glucose-6-phosphate dehydrogenase mutations in Mon and Burmese of southern Myanmar. J Hum Genet **2008**; 53:48-54.

19. Laosombat V, Sattayasevana B, Janejindamai W, et al. Molecular heterogeneity of glucose-6-phosphate dehydrogenase (G6PD) variants in the south of Thailand and identification of a novel variant (G6PD Songklanagarind). Blood Cells Mol Dis **2005**; 34:191-6.

20. Bancone G, Chu CS, Somsakchaicharoen R, et al. Characterization of G6PD genotypes and phenotypes on the northwestern Thailand-Myanmar border. PLoS One **2014**; 9:e116063.

21. Ninokata A, Kimura R, Samakkarn U, Settheetham-Ishida W, Ishida T. Coexistence of five G6PD variants indicates ethnic complexity of Phuket islanders, Southern Thailand. J Hum Genet **2006**; 51:424-8.

22. Charoenkwan P, Tantiprabha W, Sirichotiyakul S, Phusua A, Sanguansermsri T. Prevalence and molecular characterization of glucose-6-phosphate dehydrogenase deficiency in northern Thailand. Southeast Asian J Trop Med Public Health **2014**; 45:187-93.
